# Chemopreventive and Biological Strategies in the Management of Oral Potentially Malignant and Malignant Disorders

**DOI:** 10.3390/bioengineering11010065

**Published:** 2024-01-09

**Authors:** Gaia Viglianisi, Alessandro Polizzi, Cristina Grippaudo, Salvatore Cocuzza, Rosalia Leonardi, Gaetano Isola

**Affiliations:** 1 Department of General Surgery and Surgical-Medical Specialties, School of Dentistry, University of Catania, Via S. Sofia 68, 95124 Catania, Italy; gaia.viglianisi@gmail.com (G.V.); alexpoli345@gmail.com (A.P.); rleonard@unict.it (R.L.); gaetano.isola@unict.it (G.I.); 2 Head and Neck Department, Università Cattolica del Sacro Cuore, Fondazione Policlinico Universitario A. Gemelli IRCCS, Largo A. Gemelli 8, 00168 Rome, Italy; 3 Department of Medical and Surgical Sciences and Advanced Technologies “GF Ingrassia” ENT Section, University of Catania, Via S. Sofia 68, 95124 Catania, Italy; s.cocuzza@unict.it

**Keywords:** bioengineering, chemoprevention, oral squamous cell carcinoma, oral potentially malignant disorders, polyphenols, immunomodulatory therapies

## Abstract

Oral potentially malignant disorders (OPMD) and oral squamous cell carcinoma (OSCC) represent a significant global health burden due to their potential for malignant transformation and the challenges associated with their diagnosis and treatment. Chemoprevention, an innovative approach aimed at halting or reversing the neoplastic process before full malignancy, has emerged as a promising avenue for mitigating the impact of OPMD and OSCC. The pivotal role of chemopreventive strategies is underscored by the need for effective interventions that go beyond traditional therapies. In this regard, chemopreventive agents offer a unique opportunity to intercept disease progression by targeting the molecular pathways implicated in carcinogenesis. Natural compounds, such as curcumin, green tea polyphenols, and resveratrol, exhibit anti-inflammatory, antioxidant, and anti-cancer properties that could make them potential candidates for curtailing the transformation of OPMD to OSCC. Moreover, targeted therapies directed at specific molecular alterations hold promise in disrupting the signaling cascades driving OSCC growth. Immunomodulatory agents, like immune checkpoint inhibitors, are gaining attention for their potential to harness the body’s immune response against early malignancies, thus impeding OSCC advancement. Additionally, nutritional interventions and topical formulations of chemopreventive agents offer localized strategies for preventing carcinogenesis in the oral cavity. The challenge lies in optimizing these strategies for efficacy, safety, and patient compliance. This review presents an up to date on the dynamic interplay between molecular insights, clinical interventions, and the broader goal of reducing the burden of oral malignancies. As research progresses, the synergy between early diagnosis, non-invasive biomarker identification, and chemopreventive therapy is poised to reshape the landscape of OPMD and OSCC management, offering a glimpse of a future where these diseases are no longer insurmountable challenges but rather preventable and manageable conditions.

## 1. Introduction

**Figure 1:**
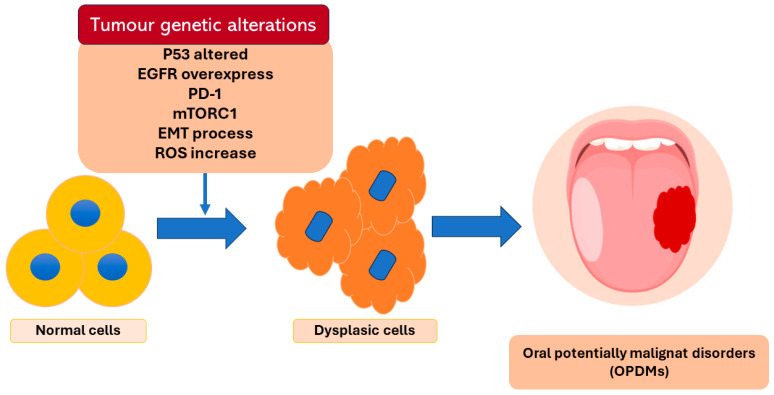
Description of some of the genetic alterations at the base of the epithelium dysplasia transformation.

Oral cancer is the sixth tumor by incidence that affects the world population and the diagnosis of around 90% of the cases is oral squamous cell carcinoma [1]. Oral squamous carcinoma is an aggressive type of cancer that gives the patients a survival rate of less than 50% at 5 years [2,3]. Smoking tobacco and the abuse of alcohol are the main risk factors for oral cancer development [4]. Along with these two risk factors, there are precancerous lesions called oral potential malignance disorders (OPMDs) that include oral leukoplakia, erythroplakia, lichen planus, and submucosal fibrosis [5]. The oral epithelium malignant transformation is the result of various mechanisms that require time for the clinic manifestation (Figure 1). The epithelial transition is not clinically visible, and it begins to be visible in the form of OPMDs [6]. 

These oral precancerous lesions are frequently asymptomatic, so most of the patients do not notice them. Consequently, patients arrive at the clinic observation when carcinoma is just probably present. Therefore, for the clinician, there are limits related to the impossibility of distinguishing premalignant oral lesions from oral cancer in situ for similar clinical manifestations [7]. In these cases, surgery, radiotherapy, and chemotherapy are the traditional treatments that are usually used. Sometimes the tumor can extend into accessible anatomical areas and the use of surgery is not always possible. Then, in these cases, surgery is frequently associated with radiotherapy or chemotherapy. All three previous treatments determine important side effects for a patient’s quality of life [8,9]. Additionally, even if the tumor can be removed with traditional therapies the survival rate after 5 years is still lower, despite the important progress achieved in the oncology field in the last three decades [10]. The survival rate for oral squamous cell carcinoma is still around 50%, and the risk of recurrence is high. Another type of treatment is the reduction of risk factors like quitting smoking, but it is not immediate [11]. For these reasons, prevention plays a fundamental role in contrasting tumor development and increases survival rates. The introduction of chemopreventive strategies in the oral cancer field aims to intercept the presence of an altered oral epithelium to suppress the possible malignant progression. Cancer chemoprevention is defined as the administration of a synthetic, natural, or biological agent to reverse, suppress, or prevent either the initial phases of carcinogenesis or the progression of premalignant cells to malignancy. Over the past ten years, there has been a significant shift in the paradigm for creating new chemopreventive drugs. Currently, agents undergo thorough preclinical mechanistic testing before beginning clinical trials, and there is a greater emphasis on identifying biomarkers of activity that can be utilized as early indicators of efficacy [12]. Various preclinic, animal studies, in vitro studies, and clinic trials, have been conducted over the years but there are still no existing guidelines for their use.

This review aims to describe the chemoprevention strategies that have been developed over the years that could be used for the treatment of oral potentially premalignant disorders and oral squamous cell carcinoma.

## 2. Oral Squamous Carcinoma and Oral Potentially Malignant Disorders

Oral cancer is a type of cancer that takes part in the group of head and neck cancers. Oral squamous cell carcinoma (OSCC) is the most common oral cancer that represents 90% of the other oral, head and neck cancers related with others oral diseases [13,14]. It is a malignant epithelial neoplasm characterized by the presence of keratin or intercellular bridges in the oral epithelium [15]. OSCC can be observed in all parts of the oral cavity [16]. The tongue, the buccal mucosa, and the lips are the typical sites where it is most frequently encountered [17]. Tobacco and alcohol are the two main risk factors of OSCC. 75% of oral cancer cases are related to tobacco smoking [4], in fact, smokers have a five times greater risk of oral cancer evolving than nonsmokers [18]. Tobacco smoke includes not less than 70 carcinogens and cancer-promoting essences [19]. The carcinogen benzo[a]pyrene (BaP) is the main constituent of cigarettes. The metabolization of BaP by cytochrome P450 enzyme allows the production of an active carcinogenic substance that can obstruct the DNA replication mechanism [20], causing cytotoxicity, teratogenicity, genotoxicity, immunotoxicity, mutagenesis, and carcinogenesis [20,21]. Alcohol consumption plays an important role in the evolution of OSCC due to its properties: (a) changes the oral mucosa permeability causing the increase of the carcinogen’s substances, (b) disintegrates the epithelium’s lipids causing the atrophy and the decrease of the mucosa layer, (c) alters the DNA synthesis and the repair mechanisms due to the acetaldehyde metabolism, (d) reduces the physiological carcinogen clearance altering the salivary gland activity [22,23,24,25].

Human papillomavirus infection, in particular HPV type 16, is a secondary risk factor of OSCC that affects less than 15% of the worldwide cases [26,27]. HPV type 16 in the human cell causes the synthesis of two oncoproteins called E6 and E7. These two oncoproteins are capable of altering the physiological function of p53 and RB1 increasing the possibility of causing lesions in the oral epithelial cells [28,29,30].

**Figure 2:**
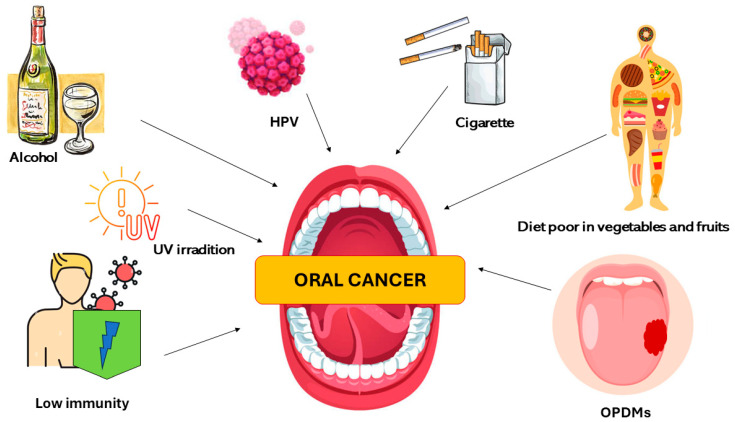
Description of the major oral cancer risk factors.

A diet poor of vegetables and fruits was seen to be another risk factor that can increase the susceptibility to develop OSCC. This is related to the fact that these foods contain phytochemicals (carotenoids, antioxidants, vitamins, phenols, terpenoids, steroids, indoles, and fibers) that are substances able to contrast the risk of developing oral pathologies [31,32]. Immune conditions (including people using immunosuppressant drugs for organ transplants), occupational exposures (UV radiation), environment pollutants, microorganisms, and genetic diseases (Fanconi anemia, dyskeratosis congenita, and Bloom syndrome) are further oral cancer risk factors [33,34,35,36]. The oral potentially malignant disorders (OPMDs) are added to the risk factors of OSCC (Figure 2).

In 2017, the World Health Organization (WHO) defined oral potentially malignant disorders (OPMDs) as a group of oral mucosal conditions with a high risk of malignant progression [37]. This new category was born because it was documented that the oral tissue affected by these conditions later evolved into OSCC. Additionally, the red and white tissue alterations that characterize these conditions can be present simultaneously in the border of the frank OSCC tissue [38,39,40]. The term “potentially malignant” underlines that not always the altered oral mucosa affected by these conditions will necessarily evolve in oral carcinoma. This term means that one of the OPMDs increases the susceptibility to the development of OSCC anywhere in the oral cavity [5,41]. The precancerous lesions (leukoplakia, erythroplakia, and proliferative verrucous leukoplakia) and the precancerous conditions (lichen planus and submucosal fibrosis) [42] take part in the OPMDs group. Leukoplakia is the most common precancerous oral lesion that affects 1–4% of the population [37,43]. Clinically it appears as white with rugose, granulomatous, or warty and nodular lesions, similar to lichen lesions [37,44,45]. In front of the leukoplakia lesions, no other diagnosis can relate the oral lesions to other causes. Leukoplakia shows a variable percentual risk of malignancy transformation from 1.5% to 34%. It is important to say that leukoplakia lesions can be homologous, and the risk of malignancy transformation is related to this clinic’s appearance. There is a non-homologous rare leukoplakia lesion called proliferative verrucous leukoplakia. It has the highest risk of oral cancer development which is from 63.3% to 100% [5]. The clinical aspect of the proliferative verrucous leukoplakia starts from a flat white lesion and develops into a verrucous and multilocular white lesion. The adjective “proliferative” underlines its characteristic of gradual extension in the near area [46,47].

Erythroplakia is another precancerous oral lesion that affects 0.02% to 0.83% of the population [48,49]. Clinically, it appears as a red spot with or without nodules, erosions, and granules. As for leukoplakia, no other diagnosis can be done [48]. For all the precancerous lesions and precancerous conditions, the diagnosis is only histological after the tissue biopsy analysis. The risk of malignancy development erythroplakia lesion is 14% to 50% [49].

Lichen planus is an oral condition characterized by the presence of chronic inflammation of the mucosa. It affected from 0.1% to 4% of the population [50]. It is an oral premalignant condition that clinically manifests itself as a bilateral white network spot [51]. Oral submucous fibrosis is the other condition that takes part in the OPMDs group. Oral submucous fibrosis is an oral condition with a high risk of oral cancer transformation that is around 9–13%. This oral condition is characterized by the gradual transformation from functional tissue to functional due to the deposit of fibrous bands [52].

## 3. Chemoprevention

In 1976 Sporn was the first who introduce the term chemoprevention [53]. Chemoprevention relies on using different natural, synthetic, biological, and chemical substances that possess anticarcinogenic actions (reverse, suppress, and prevent carcinogenic progression) [54,55,56]. Based on the target people, cancer chemoprevention strategies are divided into two categories, primary prevention, and secondary prevention. Primary prevention aims to persuade people with high risk to develop OSCC or OPMDs by changing their habits, for instance by reducing or quitting smoking. Secondary prevention has targeted patients with OPMDs or OSCC. In these cases, the prevention aims to reduce or eliminate the possibility that an OPMD lesion can have a malignant transformation and the primary OSCC lesion can have recurrences [57]. Over the years various studies have been conducted to test chemopreventive agents. The chemopreventive agents can be administered systemically, locally, and by nanoparticle delivery systems. Each of these types of administration has been shown to possess efficacy but also limits. The chemopreventive agents show many mechanisms of action against tumor cells. They can suppress the initial phase of the tumor cell transformation, operate on the altered genome induce apoptosis, and modulate the metabolic activities of the tumor cell arresting it [58].

A chemoprotective agent must possess no toxicity, show efficacy in many parts of the human body, be cheap, be orally administered, be acceptable for human uses, and be secure in its mechanisms of action [59]. The proprieties that chemopreventive agents possess allow their use for the treatment of oral precancerous lesions, but also in the early phases of the OSCC establishment. Then, chemopreventive agents can be used as a method of secondary and tertiary prevention. However, studies conducted in oral chemoprevention are still in their primordial phase and the pathogenetic mechanisms underlying oral dysplasia and carcinogenesis are not fully clarified. Consequently, most of the agents evaluated to date act predominantly with anti-oxidant, anti-inflammatory, and/or oncolytic mechanisms [60].

## 4. Phytochemical Compounds as Chemopreventive Strategy

Phytochemical compounds can be used in the chemopreventive strategies for the treatment of OSCC and OPMDs. As mentioned before, one of the factors that increase the risk of developing OPMDs and OSCC is following an incorrect diet. In fact, many studies have shown that a diet full of vegetables and fruits is associated with a low risk of premalignant and malignant lesion development [61,62,63,64,65,66,67]. Given that, the possibility of using phytochemicals contained in natural food as chemoprevention has caught the academic community’s interest (Table 1). Moreover, several researchers have found that the natural compounds present in food and drinks have no side effects [68]. Classifying phytochemicals into polyphenolic compounds, carotenoids, phytosterols, nitrogen compounds, and organosulfur compounds is possible.

### 4.1. Polyphenolic Compounds

**Figure 3:**
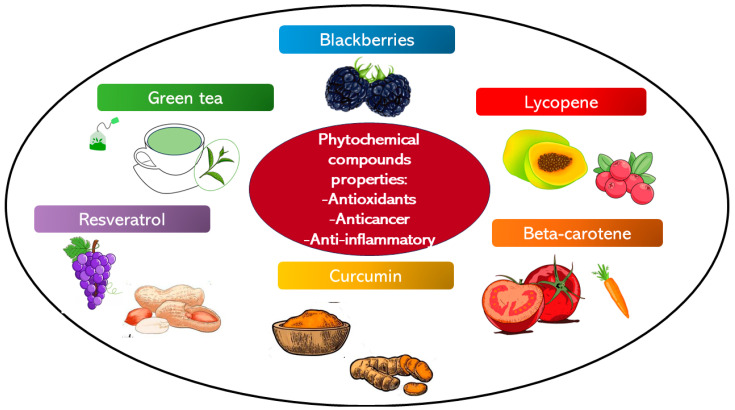
The illustration regroups the phytochemical compounds with antioxidant, anti-cancer, and anti-inflammatory properties, which have been studied as chemopreventive agents.

This group of phytochemical agents possesses antioxidant, anti-inflammatory, and anti-carcinogenesis properties (Figure 3). Related to the anti-carcinogenesis propriety these agents inhibit different phases of the cancer growth from the beginning to the progression. Polyphenolic agents can induce oral cancer cell death reducing the progression, invasion, and metastasis of the tumor [69,70,71]. Polyphenolic substances can be divided based on their chemical structure into flavonoids and non-flavonoids [72].

#### 4.1.1. Green Tea

Green tea (*Camellia sinensis*) is a component of the class of polyphenolic agents. It is normally consumed as a beverage in water. Green tea is mainly composed of four polyphenols called Epicatechin (EC), epigallocatechin (EGC), epicatechin-3-gallate (ECG), epigallocatechin-3-gallate (EGCG) [73]. Researchers have seen that green tea protects from oral-digestive cancer development, and it possesses inhibitory properties against the progress of oral precancerous lesions [74]. The main constituent of green tea that expresses many of the anti-carcinogenesis actions is EGCG. EGCG can regulate the expression of the epidermal growth factor receptor (EGFR) [75,76], the NF-κB and AP-1 factors reducing the tumoral cell’s proliferation and migration [63]. In Irimie’s study, ECGC has been shown to cause tumor cell death inducing apoptosis and autophagy [77]. The polyphenols that make green tea possess antioxidant properties. Therefore, from numerous investigations other anti-carcinogenesis mechanisms of ECGC have emerged. In a study conducted by Koh et al. [78] ECGC was administered in mice affected by squamous cell carcinoma. In the mice tested, there was a suppression of the cancer progress and invasion [78]. In an in vivo study, a mix of tropical green tea was orally and topically administered in patients affected by oral leukoplakia. The patients were treated for 6 months. After this period 40% of the patients tested showed a reduction of the oral lesion size compared to the control group (not treated). The histological analysis results were related to the significative reduction of cell proliferation [79]. In a study conducted by Gao et al. [80] another biological antitumor target against oral cancer of ECGC has emerged. In the in vitro study ECGC was administered in different concentrations in multiple human tongue carcinoma cell cultures. From this experiment has emerged that ECGC has a dose-dependent effect in decreasing EGFR expression. The same effect of ECGC against EGFR was seen against the hexokinase 2 (HK2). HK2 is an important enzyme that allows to start of glycolysis. Glycolysis is fundamental for the beginning of tumor development and progression. The authors have concluded that HK2 could be a new target for ECGC in preventing and treating human tongue carcinoma [80]. In two clinical trials, people who drink more than 10 cups of green tea daily have less risk of developing cancer than those who drink 3 or fewer cups. Furthermore, smokers who consume tea extract (2000–2500 mg/day) for 4 weeks have shown a decrease in DNA damage in oral squamous cells [81,82]. In a study conducted by Neetha et al. [83] the effect of the administration of curcumin and green tea in patients affected by OPMDs for 3 months was evaluated. From the histologic analyses of the tissue of the group of patients that received the combination of the two phytochemical agents, there was a high clinical response and improvement of the tissues. Moreover, in the group tested with the mixed combination, there was a higher downregulation of p53 and proliferation markers (cyclin D1, Ki 67) compared to the other group treated with only one of the natural agents [83]. In another study, the synergy of green tea with resveratrol was evaluated. From this in vitro study a mix with different concentrations of the two natural compounds was administered in a culture of head and neck cancer cell lines. At the end of the study have emerged that there was a dose-dependency of effects based on the combination of green tea and resveratrol. The group treated with both the natural agents showed a higher tumor cell death compared to the other group treated with the single natural compounds. Additionally, this mix of compounds was tested in an in vivo tester. From this in vivo study has emerged that in the group tested with the natural agents mix there was a superior induction of apoptosis of tumor cells and a downregulation of Ki67 compared to the other groups. For these reasons, the weight of the tumor in the in vivo caves was less than the other groups treated with single natural compounds [84].

#### 4.1.2. Resveratrol

Resveratrol (3,4′,5-trihydroxy-trans-stilbene) is another phytochemical agent present in many foods, including peanuts, mulberries, chocolate, and grapes. It possesses various anti-carcinogenesis properties [85] expressed by the enzymes participating in its metabolization. It causes the tumor cells death due to its effect of induced DNA damage in them [86]. The anti-inflammatory properties of resveratrol allow it to modulate the inflammation factors that participate in tumor progression and reduce the risk of cancer development [87,88]. Despite being seen that the administration of resveratrol up to 5 g per day in healthy patients is well-tolerated [89,90] in patients affected by diseases such as diabetes or cancers is difficult to understand the correct dose [91]. In a study conducted by Lin et al. [92], the resveratrol activities against two oral cancer cell lines were analyzed [92]. From other studies, the thyroid hormone axis actively participates in different mechanisms of human cancers [93,94]. In Lin’s study, it has emerged that resveratrol downregulates the thyroid hormones modulating the activities of PD-L1 and BTLA (two checkpoint-co-inhibitor receptors expressed on the surface of the immune system cell that when overexpressed participate in the tumor cells proliferation) [92]. Two more anti-carcinogenic mechanisms of resveratrol were discovered in different studies. Resveratrol inhibits the expression of Rab coupling protein (RCP). RCP is a protein that takes part in the proliferation and invasion of tumor cells. Furthermore, resveratrol indirectly downregulates the expression of EMT (epithelial-mesenchymal transition) reducing the metastasis proliferation in OSCC [95]. As well as for other natural compounds, combining resveratrol with other agents can enhance its anti-carcinogenesis activity. In a study by Ho et al. [96], resveratrol was administrated in association with nano-diamino-tetrac (NDAT) in two oral cancer cell lines. This study has emerged that the combination of these two substances had significantly enhanced the reduction of PD-L1 expression and tumor cell proliferation compared to the group that was treated with resveratrol alone. Moreover, NDAT in the presence of thyroxine has improved the anticarcinogenic activity of resveratrol by inhibiting a cell signaling transduction operated by SATA3 [96]. In an in vitro study on oral tumor cells, the administration of resveratrol and quercetin has been shown to induce oral tumor cell death due to DNA damage, PARP1 modification, and the upregulation of Bax. Additionally, both substances have arrested phase S of the cell’s cycle and cell’s growth allowing the inhibition of tumor cell proliferation [97].

#### 4.1.3. Blackberries

Blackberries are a fruit rich in bioactive agents including phenolic compounds, vitamins, and minerals. Different studies on various tumors have emerged that blackberries possess many anti-carcinogenesis activities [98]. In oral cancer, blackberries have been shown to induce tumor cell apoptosis [99] and modulate the pro-inflammatory factors that contribute to cancer development [99,100]. Furthermore, blackberries can modulate the cell metabolism regulating the expression of genes and metabolites related to the glycolytic pathway [101]. In a study conducted on rats affected by oral epithelial cells, the administration of blackberries showed a reduction in the cell’s DNA damage. In particular, these DNA damages were caused by the dibenzo-[a,l]-pyrene (DBP), a primary metabolite of a carcinogen component of tobacco [102,103]. Many researchers investigated the possible use of blackberries for the treatment of OPMDs. In the study conducted by Shumway et al. [104] 0.5 g of local blackberry gel was administered in patients affected by oral intraepithelial neoplasia. The gel was applied four times per day for a total of 6 months of treatment. After this time, a histological analysis was performed and compared with the initial biopsy. There was a reduction of the oral lesions dimension, an improvement of the histological grade, and a decrease in the initial loss of heterozygosity indices (LOH) [104]. In another study, a gel containing 0,5 g of blackberries was administered to a group of patients affected by oral intraepithelial neoplasia. The control group was treated with a gel with a placebo. The treatment was performed for 12 weeks. At the end of the study, the group tested showed a reduction of the size and an improvement of the histological grade and LOH compared to the control group which showed an increase of the size and no improvement in the histological characteristics [105].

### 4.2. Carotenoids

Carotenoids are a group of pigments that belong to the tetraterpenes family. The carotenoid compound is drawn from the 13-carotene, the precursor of the vitamin A [106,107]. Carotenoids are present in the organisms that do photosynthesis, and they protect them from the possible damage caused by the sun’s rays. Carotenoids provide the typical yellow, orange, and red colors to fruits and flowers. It is possible to classify carotenoids into provitamin A and non-provitamin A substances [108]. α-carotene, β-carotene, lycopene, β-cryptoxanthin, lutein, and zeaxanthin are the main carotenoid compounds assumed in the dietary [109]. The carotenoid compounds are classified into carotenes (α-carotene, β-carotene, and lycopene) and xanthophylls (β-cryptoxanthin, lutein, and zeaxanthin) based on their chemical structure [110]. These compounds possess anti-inflammatory and antioxidant functions. A large consumption of carotenoids in diet has been shown to reduce the risk of developing cancer [111]. Carotenoids of the provitamin A group perform actions on cell proliferation and differentiation like vitamin A [112] (Table 1).

#### 4.2.1. Beta-Carotene and Vitamin A Derivates

Beta-carotene is the main carotenoid group component. It is present with its red-orange-yellow colors in various fruits and vegetables including tomatoes, carrots, and beans. Beta-carotene possesses antioxidant and anti-carcinogenic properties [113]. Beta-carotene has been shown to reduce the expression of cyclin A allowing the cell cycle arrest. Additionally, it can downregulate the activity of the anti-apoptotic BCL-2 and upregulate the expression of p53 inducing the tumor cells’ apoptosis [114]. In the oral field, different studies were conducted to find the possible use of this compound against premalignant and oral cancers. In a study performed on patients affected by leucoplakia beta-carotene and vitamin C were administered for 1 year. At the end of the study, the authors concluded that the administration of beta-carotene and vitamin C had no effects on the clinical improvement of the leucoplakia lesions [115]. In another study conducted by Buajeed et al. [116], beta-carotene was administered in patients affected by atrophic and erosive oral lichen planus. Beta-carotene was given for 3 months with a dose of 15 mg daily. At the end of the study, the characteristics of the sample of the cells exfoliated have been analyzed. From these analyses, the treatment with beta-carotene reduced the number of the micronucleated exfoliated cells, typical of the lichen planus pathology [116].

Vitamin A is a liposoluble essential vitamin that participates in the epithelium’s normal functioning. Retinoids and carotenoids derive from vitamin A, and they have been shown to possess anti-tumor properties. Retinoids decreased free radicals, induced apoptosis, and downregulated epithelial cell proliferation. Isotretinoin (13-cis-retinoic acid), derived from vitamin A, has shown promising results in the treatment of oral leukoplakia. In a study conducted by Hong et al. [117], isotretinoin was administered at 1–2 mg/kg per day in patients affected by oral leukoplakia. At the end of the study, 67% of the patients tested showed clinical response. The histological analysis has exhibited that isotretinoin had reduced the size and the grade of dysplasia in the oral leukoplakia lesions of the group tested compared to the control group which was administered a placebo. Despite these promising results, the study has shown limitations, which are the toxicity of isotretinoin and the high rate of recurrence after a few months [117]. Further investigations have been conducted to discover the nontoxic isotretinoin dose, but no one has found it [118]. Retinyl palmitate and beta-carotene, other vitamin A derivates, have shown efficacy but less toxicity than isotretinoin [119]. In a study conducted by Papadimitrakopoulou et al. [120] the efficacy of isotretinoin, retinyl palmitate, and beta-carotene in the treatment of OSCC was compared. The study results have shown that all three substances have proved effective in the treatment of OSCC, but isotretinoin possesses a higher clinical response [120]. It was proved that the association of vitamin E reduces isotretinoin toxicity [121].

#### 4.2.2. Lycopene

Lycopene is a carotenoid present in many foods including tomatoes, cranberries, papayas, peaches, grapes, apricots, watermelon, and pink grapefruits [122]. It possesses antioxidant and anticancer properties. Due to these activities, lycopene was tested for treating different oral diseases, including OPMDs and OSCC. Kumar et al. [123] after their study concluded that lycopene should be used in the first-line therapy for the treatment of oral submucous fibrosis. From an in vivo study have emerged that the administration of lycopene in mice affected by oral squamous cancer can induce the apoptosis of the oral tumor cells due to the regulation of BAX (a pro-apoptotic protein) and bcl-2 (an anti-apoptotic protein). Specifically, lycopene administration induced an increase in the ratio of Bax to bcl-2, which resulted in apoptosis of cancer cells. Additionally, lycopene administration has been shown to inhibit the epithelial-mesenchymal transition (EMT) mechanism and the PI3K/AKT/mTOR signaling pathway [124]. These two mechanisms are related to tumorigenesis, cancer progression [125,126,127,128] and metastasis [129,130]. In a study conducted on cultures of cells affected by OSCC the treatment with lycopene showed a decrease of the expression of the insulin-like grow factor 1 (IGF1) and a reduction of the cancer tissue size compared to the control group (treated with a placebo). IGF1 contributes to the carcinogenesis mechanism, allowing tumor cell proliferation. Furthermore, the efficacy of different dosages of lycopene (0.25, 0.5, 1, and 2 µM) was studied. At the end of the study, the results obtained were dose-dependent based on the lycopene quantity [131]. Another study has emerged that lycopene increasing the glutathione redox cycle inhibits tumor progression in cells affected by oral cancer [132]. Additionally, lycopene modulates the expression of connexin-43 (a gap-junction protein) and reduces the killer B1 cells in oral cancer [133]. In a study conducted by Singh et al. [134] lycopene’s efficacy was tested in treating leucoplakia. The tester groups were administered different dosages (8 mg/day, 4 mg/day) of lycopene and a placebo was administered in the control group. At the end of the study, after 3 months, in the two groups tested it were seen a reduction in the size and the hyperkeratosis of the leucoplakia lesions compared to the control group. Despite higher improvement in the clinical results, no statistical differences existed between the group administered 8 mg/day and the group tested with 4 mg/day [134].

#### 4.2.3. Curcumin

Curcumin contained in the rhizome of Turmeric (*Curcuma longa*) takes part in this class of phytochemical agents. Curcumin possesses different properties including anti-inflammatory, antioxidant, antimicrobial, immunomodulatory, and antitumor. Curcumin inhibits the activity of nuclear factor kappa B (NF-κB). NF-κB is an important factor that participates in the inflammatory process. It is known that the persistence of an inflammatory process participates in the stages of cancer establishment, for instance, tissue hyperplasia [135,136]. In fact, in different cancers, including oral cancer, the persistence of inflammation due to the NF-κB activity was seen [137]. Moreover, from an in vitro experiment curcumin has downregulated the matrix metalloprotease 2 and 9 (MMP-2 and MMP-9) and modulated the expression of E-cadherin and p53 [138]. MMP-2 and MMP-9 are enzymes that participate in extracellular matrix degradation, which is crucial for cancer cell invasion and metastasis [139]. E-cadherin is a cell adhesion molecular. Different studies have found in many cancers that the loss of E-cadherin expression is a marker of tumor invasion and metastasis progression [140,141]. From an in vitro study curcumin can reduce the expression of the two transcription factors (Twist and Snail) that caused the inhibition of E-cadherin expression [138]. P53 is one of the main tumor suppressor proteins, the p53 alteration causes metastasis and invasion progression [142,143]. In a study conducted by Kuttan et al. [144], curcumin was locally administered to 62 patients affected by oral cancers. At the end of the study, 10% of the participants showed a reduction in the dimension of the cancer lesions [144]. Cheng et al. [145] have conducted a study to evaluate the effects of the oral administration of curcumin for 3 months in patients affected by oral leukoplakia. Before and at the end of this period the biopsies of the oral lesions were made. From the histological analysis, there was an improvement in the seven cases treated [145]. In a study conducted by Tuttle et al. [146] the efficacy of curcumin in association with radiotherapy was tested for the treatment of OSCC. From this study, it has emerged that mice affected by OSCC negative to HPV treated with this combination of therapy have shown an increase in the survival rate compared to the other group [146]. The results obtained in Tuttle’s study could be related to the fact that curcumin has been shown to protect the non-tumor cells and tissue from ionic radiation during tumor radiation therapy, and it has induced radiosensitive in the oral squamous carcinoma cells [147,148]. The authors concluded by saying that the association of curcumin with radiotherapy could be used as a first-line therapy for the OSCC treatment [146].

Although the results obtained in the previous study related to the possible use of phytochemical compounds as chemotherapy there are limits. The bioavailability of these substances is one of the main limits because they are distributed in the organism and quickly removed in the liver after their administration. Additionally, natural compounds are difficult to absorb in the stomach; this is another important limit. Then, not all the substance administered arrives in the specific area where it is needed [149,150,151]. To contrast these limits, there is the possibility to use nanoparticles and lipids that can be loaded with natural compounds [149,151,152].

**Table 1:** A summary of the different phytochemical compounds with their dietary sources and mechanisms of action.

Phytochemical Compound	Substance	Dietary Source	Mechanism of Action	Reference
Polyphenolic	Green tea		Regulate the expression of the EGFR, the NF-κB and AP-1 factors, induce tumor cell death	[63,75,77]
	Resveratrol	peanuts, mulberries, chocolate, and grapes	Causes tumor cell death, performs an anti-inflammatory activity, downregulates the thyroid hormones modulating PD-L1 and BTLA, inhibits the expression of RCP, indirectly downregulates the expression of EMT	[86,87,88,92,95]
	Blackberries		Induce tumor cell apoptosis, decrease inflammatory factors, and modulate the cell’s metabolism	[99,100,101]
Carotenoids	Beta-carotene	tomato, carrots, and beans	Reduces the expression of cyclin A, downregulates the activity of BCL-2, and upregulates the expression	[114]
	Vitamin A derivates		decrease free radicals, induce apoptosis, and downregulate the epithelial cells’ proliferation	[117]
	Lycopene	tomato, cranberries, papayas, peaches, grapes, apricots, watermelon, and pink grapefruits	Regulates BAX and bcl-2, inhibits EMT mechanism and the PI3K/AKT/mTOR signaling pathway, decreases the expression of IGF1	[124,131]
	Curcumin		Inhibits NF-κB activity, downregulates MMP-2 and MMP-9, reduces the expression of Twist and Snail, modulates the expression of E-cadherin and p53	[135,137]

## 5. Immunomodulatory Agents

**Figure 4:**
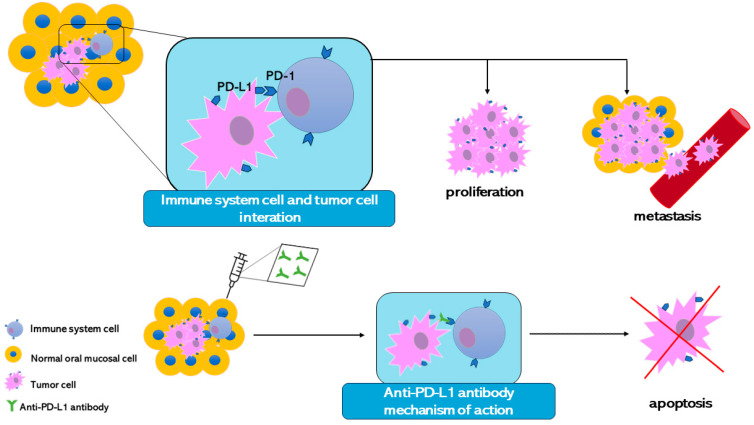
The illustration represents one mechanism that tumor cells perform to avoid immune system control. The introduction of the monoclonal antibody has allowed the development of an anti-PD-L1 antibody to contrast the tumor cells’ escape mechanism. In this way, the immune system can recognize the tumor cell and induce its apoptosis.

Through the improvement of the information about the tumor cell development mechanisms, it has been discovered that tumor cells can escape the immune system control. Normally immune cells bind surface proteins present on the cells and with this mechanism, immune cells can discover the presence of stranger antigens. Although this mechanism protects the organism, at the same time the organism can attack itself. For this reason, proteins that can turn off the immune response are linked to the organism’s cell surface. This mechanism avoids autoimmunity. Tumor cells express on their surface the same proteins to switch off the immune system allowing it to survive and proliferate. Therefore, experts are starting to study and develop new cancer therapy that targets the immune checkpoint receptors. The idea is to create a monoclonal antibody able to bind the immune checkpoints. PD-1 is one of the main immune checkpoints studied. Despite the immune system tumor cell being recognized as a stranger, it produces PD-L1, a protein that binds PD-1 and turns off the immune responses against itself [153] (Figure 4).

In the oral pathologies and cancer fields, this discovery has permitted the opening of a new type of antitumor therapy. In a study conducted by Wang et al. [154] a monoclonal anti-PD-1 antibody was tested on animals affected by oral leukoplakia. This study has emerged that blocking the interaction of PD-L1 with PD-1 has facilitated T cell activity, inducing the apoptosis of the cell that formed the oral leukoplakia lesion. The histological analysis showed that in the mice treated with the monoclonal antibody, there was a decrease (43.3%) in the grade of dysplasia of the oral leukoplakia lesions compared to the control group. The authors suggested that monoclonal anti-PD-1 could be used for the treatment of the oral precancerous lesions to contrast the malignancy development and to treat OSCC. However, evaluations are necessary before clinical use of this anti-PD-1 antibody. Furthermore, from this study has emerged that another checkpoint should be blocked. In mice treated with anti-PD-1, it was seen an increase in the activity of another checkpoint called CTLA-4. Then, using different monoclonal antibodies against PD-1 and CTLA-4 can enhance the antitumor activity. The blockade combination of these two checkpoints has improved in patients affected by melanoma [155,156,157]. In 2016 the U.S. FDA approved pembrolizumab and nivolumab, two monoclonal anti-PD-1 antibodies. After different trial studies, it was concluded that the use of pembrolizumab and nivolumab increases the survival rate in patients with advanced head and neck cancer who do not respond to platinum-based chemotherapy [158,159]. The possibility to use immunotherapy to treat oral cancer instead of the use of traditional chemotherapy allows the use of drugs with a specific molecular target reducing the side effects connected with the chemotherapy. Even though these are positive characteristics, side effects have occurred also using immunotherapy to inhibit immune checkpoints [160].

## 6. Topical Formulation

Topical formulation is another chemopreventive strategy against the malignancy development of the OPMDs. This type of administration possesses different advantages including the reduction of toxicity and systemic metabolism, the possibility to use the chemopreventive substances directly in the margins of the oral lesion, and the increase in the local absorption of the chemopreventive substances only in the area where is necessary [161]. Various studies have been conducted to find which agents can contrast the malignancy development of oral precancerous lesions. In particular, the compounds that were most tested are vitamin A derivates (isotretinoin and tretinoin) [162,163,164,165,166,167], bleomycin [168,169,170,171,172], and cyclooxygenase inhibitors [173] (Table 2). There were also natural substances like raspberries that were previously discussed.

### 6.1. Retinoids

Vitamin A derivatives are the main topical agents that have been studied to treat OPMDs. Retinoids perform their antitumor activities by binding specific receptors that are retinoid receptors (RARs) [174] and activator protein 1 (AP-1) [175,176]. RARs allow retinoids to influence the transcription or not of different genes regulating the growth and proliferation of cell mechanisms [174]. AP-1 regulates various processes, including inflammation, spread, and oncogene function [175,176]. These specific targets allow retinoids to interrupt the tumor cell growth and expansion. In the studies conducted by Piatelli et al. [166] and Tete et al. [163] the action of 0.1% of isotretinoin was tested in patients affected by oral leukoplakia. In Piatelli’s study, 10 patients were enrolled and randomly divided into two groups: the tester group and the control group (treated with a placebo gel). The local gel administration was performed for 4 months. At the end of the study, the group tested saw a clinical improvement in the oral lesion compared to the control group in which there was no variation [166]. Similar results were obtained in Tete’s study in which 15 patients were enrolled and randomly divided into two groups the tester and the control. The tester group was treated for 4 months with 3 daily local administrations of a gel composed of 0.1% isotretinoin. The outcomes of the study showed 3 complete remissions and 11 enhancements of the clinical lesions greater than or equal to 50% [163]. In another study by Scardina et al. [167] the efficacy of a gel of 0.18% isotretinoin was tested for the treatment of oral leukoplakia lesions. 40 patients were enrolled and divided into two groups: group A treated with a gel of 0.18% of isotretinoin and Group B treated with a gel of 0.05% of isotretinoin. The gels were applied two times per day for 3 months. At the end of the study in the group treated with the higher concentration of isotretinoin, there was a total regression of 85% of oral lesions [167]. Even though the use of isotretinoin has been shown to improve the clinical aspect of oral leukoplakia lesion, some patients have suffered side effects including erythema, xerostomia [167], burning tongue, dry lips, and mucositis [162,163,164,165]. As demonstrated by these previous studies the local administration of retinoids has shown efficacy in the treatment of the oral leukoplakia lesions. Despite these positive effects, some common side effects are present and for these reasons, further studies are necessary for a better understanding of the well-tolerated dose.

### 6.2. Bleomycin

Bleomycin is another agent that was tested for premalignant oral lesions therapy. Bleomycin is an antibiotic that has been shown to possess antitumor effects [177]. It was seen that bleomycin can suppress the DNA replication binding DNA synthesis enzyme [178]. Bleomycin was used to treat different cancers including head and neck cancers, lymphoma, and testicular tumor [179,180]. The systemic administration of these antitumor agents has shown toxicity, in particular for skin and lungs. For this reason, bleomycin started to be used with another type of administration, the iontophoretic [181]. In the study conducted by Hammersley et al. [170] the efficacy of the local administration of bleomycin for 15 days was tested. The study was conducted on 6 patients who at the end of the experiments, showed a reduction of the dysplasia and keratinization in the cells of the clinical oral lesions [170]. Similar results were obtained in a study in which bleomycin was applied for 3 months [171]. Wong et al. [172] used different concentrations of topical bleomycin for the oral leukoplakia lesion treatment. The study was conducted on 12 patients divided into two groups: one group was treated with 0.5% bleomycin and the second group was treated with 1.0% bleomycin. The study results have shown that clinical enhancement is dose-dependent to the bleomycin concentration. Moreover, two of the six patients treated with bleomycin at 1.0% concentration showed total remission at the end of the study. The authors concluded that increasing the topical administration over a long time could allow the total remission [172]. Similar results have been obtained in two long prospective studies conducted by Epstain et al. [168,169]. The local administration of 1% of bleomycin has reduced the grade of dysplasia allowing the regression of the oral leukoplakia lesions [168,169]. All the authors of the previous studies have underlined the importance of regular follow-up in patients affected by oral leukoplakia that is under treatment because some patients have shown the presence of carcinoma in situ despite the treatments.

### 6.3. Cyclooxygenase Inhibitors

The cyclooxygenase (COX) inhibitors are the other group of agents that have been studied as a chemopreventive strategy against the malignancy development of precancerous lesions and OSCC. This category of drugs is normally used in cases of inflammation because they are a nonsteroidal anti-inflammatory drug. The idea to use COX inhibitors as antitumor drugs in the oral field was born when Panjie et al. [182] reported that with this drug, there were clinical effects on patients affected by head and neck tumors [182]. Additionally, in other studies, it has been seen that the administration of this category of drug in patients affected by lung, colon, esophageal, and breast tumors could reduce the tumor risk [183,184,185]. Two types of COX are expressed in the human body: COX-1 and COX-2. COX-1 is generally expressed for the human body’s physiological mechanism, while COX-2 is expressed in the case of inflammation and proliferation due to incentives. The COX-2 activity determines the increase of prostanoid production, and this overexpression takes part in the carcinogenesis mechanisms [186]. These mechanisms form the basis of the multi-phases of the OSCC development. The use of celecoxib, a COX inhibitor, has allowed the COX-2 and prostanoid levels reduction in patients affected by oral premalignant lesions [187]. In the study conducted by Mulshine et al. [173] the efficacy of a mouth rise composed of 0.1%/10 mL of ketorolac was tested in patients with oral leukoplakia. The study was conducted on 57 patients randomly divided into two groups: one group was treated with an oral rinse with ketorolac and the other group with an oral rinse with a placebo. The oral rinses were administered two times in 3 months. At the end of the study, the clinical size of the oral lesions and the histological analysis were analyzed and compared with the time 0 (before the experiment). The results obtained showed that there were no differences between the two groups. The authors concluded that using this oral rinse with a COX inhibitor was inefficient in the oral leukoplakia treatment [173]. Similar results and conclusions have been obtained in the study achieved by Papadimitrakopoulou et al. [188]. The previous results are probably related to the two main limits of the COX inhibitors: low absorption and bioavailability. Additionally, from other studies, it was seen that the prolonged use of COX-2 inhibitors can cause cardiotoxicity [189] and gastrointestinal problems [190] To contrast these limits researchers have studied the possibility of using nanoparticles as vehicles of COX inhibitors [191,192]. The nanoparticle system allows to carrying of the drug to the target site reducing the side effects of the systemic administration and increasing the local concentration. For these reasons, future studies are needed to test the use of nanoparticles as vehicles for COX inhibitors in the treatment of precancerous and cancerous lesions of the oral cavity.

### 6.4. Photodynamic Therapy

Photodynamic therapy is another chemopreventive strategy against oral cancer and precancerous oral lesions. This therapy is based on the sensitivity of particular substances (photosensitizers) to a specific range of light. The interaction of these two substances caused oxygen reactions, creating reactive oxygen species (ROS) in the tissue exposed. The possibility of administering the photosensitizer and irradiating small areas of tissue reduces the side effects and increases the bioavailability and efficacy [193]. The ROS produced during the irradiation of the tissue performs antitumor activities, killing tumor cells, damaging the cancer’s vascular tissue, and stimulating the immune system against the tumor [194]. In the tissue that had to be treated, the inactivated photosensitizer is administered topically or with an injection. 5-Aminolevulinic acid (ALA) is the most used inactivated photosensitizer [195,196]. Different clinics have tried to study photodynamic therapy’s use in treating oral precancerous lesions. In a study conducted by Lin et al. [197] the efficacy of the photodynamic therapy was tested to treat oral erythroleukoplakia and oral verrucous hyperplasia. The study was conducted on 40 patients diagnosed with oral erythroleukoplakia and 40 patients diagnosed with oral verrucous hyperplasia. The ALA was administered with a local gel solution at 20% with a composition able to resist saliva dilution. The administration of ALA and the photo irradiation with a diode laser were applied once a week for all the patients. The clinical resolution of the oral lesions was seen after around three treatment sessions. The authors concluded that this therapeutic strategy is the greatest option for the treatment of oral precancerous lesions [197]. Similar results were obtained in the study performed by Yu et al. [198], in which the efficacy of the photodynamic therapy was analyzed for the treatment of oral verrucous hyperplasia. In the study, 36 patients diagnosed with oral verrucous hyperplasia were enrolled. The ALA gel preparation was administered in all patients, and after one hour and a half, the oral lesions were irradiated with a red light using an LED. This protocol was performed once a week up to the resolution of the lesions. From the study results, the patients enrolled have shown a regression of the oral lesions after fewer than seven appointments. The authors underline that the period of treatment is based on the size, the range of the oral lesion color, and the thickness of the keratin surface [198].

### 6.5. P53 Inhibitor

In about 50% of the patients affected by head and neck cancers, there is an alteration of the p53 gene that caused its inactivity. P53 performs a crucial role in the correct functioning of the cell, and it is called “the guardian of the genome” [199,200,201]. The modification of the p53 determines the beginning of the primary stage of carcinogenesis. The p53 inactivity caused the uncontrolled proliferation of cells with low differentiation [202]. As a result of this discovery, academics have begun to search for an antitumor substance that targets the mutant p53. This category of drug was developed to arrest tumor proliferation. This category of drug performed different mechanisms of action that are: delivering the wild type of the p53 gene into the tumor cell using an adenovirus, inducing the apoptosis in the tumor cell with p53 mutated using an adenovirus, and correcting the mutated p53 through the delivering of small particles. These are the p53 inhibitor mechanisms of action used in oncology therapy to reduce the lack of responses of resistance tumors and recurrence to traditional therapy [203]. In the chemopreventive field, it was seen that using the p53 inhibitors associated with traditional chemopreventive strategies there was an enhancement of their efficacy [204]. From different studies, the attenuated adenovirus named ONYX-015 was able to cause the death of the cells with mutant p53 [204,205,206]. This attenuated adenovirus does not have the specific protein that allows it to replicate itself in human cells. In this way, this adenovirus has to target the cell with p53 inactivated in which it can replicate itself causing the death of the tumor cell. In normal cells the absence of the specific protein and the normal activity of p53 cause the impossibility of replicating the altered adenovirus [204]. Based on these discoveries, an oral rinse composed of ONYX-015 was tested in the study conducted by Rudi et al. [207]. The study was conducted on 22 patients affected by oral leukoplakia and erythroplakia divided into three groups. In the first group, the oral rinse was administered once a day for 5 days to repeat every month for a maximum of 12 cycles. In the second group, the oral rinse was administered once a week for 6 months. In the third group, the oral rinse was administered daily for 5 days and after these days weekly for more than 1 month. The histological results have shown that 37% of the patients treated had the dysplasia resolution. Despite these results the long-term efficacy there was not seen [207]. Similar conclusions have emerged from the study of Li et al. [208] in which the attenuated adenovirus was administered with local infiltrations [208]. The p53 mutation, present in the oral tumor cell, can be a specific target to induce apoptosis, arresting tumor proliferation.

**Table 2:** This table summarizes the different types of chemopreventive topic agents and their mechanisms of action against the altered cells.

Substance	Mechanism of Action	Reference
Retinoids	Bind RARs and AP-1 regulating the expression of genes related to cell growth and proliferation	[174,175,176]
Bleomycin	Suppress the DNA replication binding DNA synthesis enzyme	[178]
Cyclooxygenase inhibitors	Permitted the reduction of the production of COX-2 and prostanoids leading to the reduction of the inflammatory state	[187]
Photodynamic therapy	The local administration of a photosensitizer (ALA) after its activation with light causes the production of ROS that provokes tumor cell death	[193,194]
P53 inhibitor (ONYX-015)	Bind the altered p53 gene, causing tumor cell death	[204,205,206]

## 7. EGFR Inhibitor

Epidermal growth factor receptor (EGFR) is important for the physiological proliferation of epithelial cells. In many cancers, including head and neck cancers, there is an alteration of the EGFR expression that causes an increase in cell proliferation and the resistance to apoptosis, allowing invasion and metastasis processes [209]. For this reason, various studies have been conducted to find a target drug able to inhibit EGFR. A monoclonal antibody called cetuximab was developed to inhibit the EGFR activity. It was authorized by the U.S. FDA in 2006. Cetuximab has been shown to prevent epithelial cell proliferation [210,211,212,213,214]. Additionally, it was studied that the overexpression of EGFR in oral cell cancers is related to less radiation sensitivity [215]. The administration of cetuximab and radiotherapy has shown higher efficacy in preventing the proliferation of oral tumor cells. This is related to the fact that cetuximab can increase the radiosensitivity of the tumor cells [210,211,212]. Therefore, the administration of cetuximab associated with the radiotherapy treatment has increased the survival rate at 5 years compared to the survival rate at 5 years after the use of radiotherapy alone (45.6% vs. 36.4%) [214]. Different studies have underlined the efficacy of cetuximab for the treatment of OSCC in association or not with radiotherapy. Despite that, cetuximab is administered intravenously and its extended use must be limited [216] and targeted for secondary chemoprevention. In a study conducted by Ohnishi et al. [217] it was discovered that long use of cetuximab can cause a loss of efficacy in treating precancerous and cancerous oral lesions. Further studies are necessary to be performed for a better knowledge of the efficacy of cetuximab over the long period of its administration.

The alteration of EGFR is also present in the precancerous lesions and the normal tissues [218]. In the study conducted by William et al. [216] the efficacy of erlotinib was investigated in the treatment of oral leukoplakia [216]. Erlotinib is a tyrosine kinase inhibitor able to bind EGFR suppressing its activity [219]. In the trial made by William et al. [216] 379 patients affected by oral lesions with a high malignancy diagnosis were enrolled and divided into two groups. Patients of the first group were treated with 150 mg of erlotinib and patients of the second group were treated with a placebo for 12 months. At the end of the study, no statistically significant change in the survival rate was seen between the group treated with erlotinib and the placebo group. From the results obtained, the authors concluded that in the long period, erlotinib had no efficacy in treating oral leukoplakia lesions with a high risk of malignancy evolution. However, William’s study has underlined the importance of EGFR in the diagnosis and prognosis of precancerous lesions [216]. Even though erlotinib reduces the activity of EGFR overexpress, the administration of this drug has caused different side effects. The development of rash is the main side effect. This is because the EGFR inhibitor settles in the lesion in which there is a high expression of EGFR. This side effect of erlotinib is the only reason researchers are discouraged from increasing its dosage [220,221]. Different studies conducted on animals and in vitro showed that the combination of erlotinib and celecoxib could contrast the OSCC tumor progression [222,223,224]. Consequently, Saba et al. [225] have performed a study in which the efficacy of the combination of celecoxib and erlotinib was investigated in the treatment of advanced precancerous lesions. For the study, 36 patients with different grades of oral leukoplakia or with carcinoma in situ were enrolled. Patients have received 400 mg of celecoxib and three different doses of erlotinib 50 mg, 75 mg, and 100 mg, during the 6 months of treatment. At the end of the study, the histological analysis showed a decrease in EGFR expression. Despite this promising result, different patients have exhibited the main side effect related to the use of erlotinib which is rash [225]. Due to the applicant rash in patients and the few studies conducted, further research is necessary to better understand the use of EGFR inhibitors as chemopreventive agents against OSCC and OPMDs.

## 8. Metformin

As mentioned previously in this article glycolysis is fundamental for the beginning and maintenance of the tumor cells’ activities. Given that, various investigations have been conducted to understand if the use of anti-diabetic drugs has efficacy in the OSCC and in the OPMDs chemoprevention. Metformin is the most common anti-diabetic drug used in the world for the treatment of type 2 diabetes. Some studies have discovered that diabetic patients who assumed metformin for diabetic therapy have shown a lower risk of developing cancer [226,227,228]. This discovery is related to the fact that metformin can control the functioning of the mTORC1 pathway [229]. Additional studies have shown how the mTORC1 pathway actively contributes to developing head and neck cancers and precancerous oral lesions [230,231,232]. Metformin causes tumor cell apoptosis suppressing the mTORC1 pathway [233,234,235,236,237]. Moreover, metformin has another antitumor activity that is performed by the activation of liver kinase B1 (LKB1)–5′ AMP-activated protein kinase (AMPK), a different cell’s pathway [238,239,240]. The possibility of using metformin as a chemopreventive agent is also validated by one investigation in which the diabetic patients affected by OSCC that assumed metformin showed the lowest risk of recurrences and metastasis with a better prognosis [241]. It is also well known that the use of metformin has a very low toxicity for humans [242]. In the study conducted by El-Zalabany et al. [243] the efficacy of metformin as a chemopreventive agent was investigated for treating OPMDs. For the study, 40 patients affected by leukoplakia and lichen planus without diabetes were enrolled. The patients were divided into the tester group, which administered 500 mg of metformin hydrochloride once a day, and the control group treated with a placebo. At the end of 3 months, the group tested showed a reduction of the oral lesions size and the grade of epithelium dysplasia compared to the control group in which the lesions were bigger than in the beginning. From the historical analysis has also emerged that metformin has allowed the decrease of Cyclin-A2 (a regulator of cell proliferation), miRNA-31, and miRNA 210 concentrations (tumor cancer biomarkers). Based on the results obtained, the authors of this investigation suggested the use of metformin to prevent the malignant evolution of OPMDs [243]. In another study, metformin was administered in patients affected by leukoplakia or erythroplakia for 3 months. Metformin was assumed by patients with different dosages in the three months of therapy: first month 500 mg, second mouth 1000 mg, and the last month 2000 mg. From the results obtained, it has emerged that 60% of the patients treated have shown clinical responses. The histological analysis indicated that metformin has performed antitumor activity, causing a statistically significant reduction in the proliferation of dysplasia cells [244]. A combination of different substances is possible to be used as a chemopreventive strategy. In the study conducted by Siddappa et al. [245] the efficacy of the use of metformin and curcumin was tested for the treatment of ODPMs. The study was conducted on mice affected by OSCC divided into two groups. The tester group received 5 mL of water daily which contained curcumin at 14.3 µg/mL and metformin at 5 mg/mL, while the control group received water with a placebo. At the end of the study, the group treated with the combination of curcumin and metformin showed a decrease in the oral lesions size compared to the control group. Additionally, the treatment has enhanced the animals’ survival rate [245]. For the promising results obtained in these and other studies, researchers underline the potential that metformin can be performed as a chemopreventive agent in the treatment of OSCC and OPMDs.

## 9. Vitamin E

Vitamin E is a vitamin that performs antioxidant activities. It is present in different common foods including margarine and plant oils. Alpha-tocopherol is the main ingredient of vitamin E. Vitamin E has been proposed as a chemopreventive agent due to the antitumor activity performed as an antioxidant compound. In the study conducted by Benner et al. [246] the efficacy and the limit of vitamin E were investigated in the oral leukoplakia lesions treatment. For the study, 34 patients were enrolled and were treated with two daily administration of 400 IU of vitamin E for 6 months. At the end of the study, 46% of the patients showed clinical improvement and 21% showed histological enhancement [246]. In another study, the efficacy of a mix of different antioxidant supplements that were beta-carotene, ascorbic acid, and alpha-tocopherol was investigated for the oral leukoplakia treatment. The study was conducted for 9 months and after that 55% of patients saw clinical improvement. Additionally, even in patients who did not have reduced risk factors like tobacco and alcohol clinical improvement was noted [247]. The daily limit dosage of vitamin E for men and women was discovered to be 8 mg and 10 mg. A higher dose has been shown to cause a problem, increasing the mortality risk, and this has to be prevented [248]. Due to the few studies, further investigations must be conducted for a better understanding of the efficacy of vitamin E as a chemopreventive agent.

## 10. Chemopreventive Agents Loaded on Nanoparticles

In the last years, the introduction of nanoparticles has increased researchers’ interest in different medical fields. Nanoparticles can lead to chemical substances bringing them into a target area. They also allow for to reduction of the common side effects typically of the systemic administration of drugs. Due to the numerous advantages that nanoparticles possess; researchers have begun testing their use with chemotherapeutic substances. Following are some examples of studies conducted about chemopreventive agents and delivery systems. In the study conducted by Maller et al. [105] it was formulated and tested a mucoadhesive berry gels. The absorption and penetration capacity of the substances within the oral tissues was analyzed. From the analyses conducted in this study, the authors concluded that their mucoadhesive gel formulation was well adapted for oral mucosa penetration and absorption [105]. Chang et al. [249] have created a delivery system to reduce the low solubility of curcumin composed of PLGA nanoparticles loaded with curcumin. This nano delivery system was tested on an in vitro culture of oral tumor cells cisplatin resistant. From the study have emerged that nanoparticles allow curcumin to penetrate more easily into the cells increasing its concentration. Moreover, the nanoparticle with curcumin injected into oral tumor cells has been shown to stimulate apoptosis activating the caspase cascade mechanism [249]. Similar results were obtained in the study of Mazzarino et al. [250] in which curcumin was loaded with chitosan. From this study, it was seen that chitosan had permitted an increase of the curcumin penetration into the culture of oral tumor cells. These had determined the reduction of the oral tumor cells viability and the apoptosis [250] These studies show some of the advantages that nanoparticle systems can bring when combined with traditional chemopreventive agents. Further studies concerning the use of nanoparticles with various chemopreventive agents on a larger population are needed to verify the efficacy of their use in the treatment of preneoplastic and neoplastic lesions of the oral cavity.

## 11. Conclusions

Although the primary prevention, the prevalence and the incidence of OPMDs and OSCC are high. Given the necessity to develop new strategies, chemoprevention was born to halt OPMDs’s malignant transformation and oral cancer progression. The studies conducted until today have been performed on a small sample of the population, and many of them have only been conducted on cell cultures or animals. Moreover, the partial efficacy and possible side effects are other limits of the compounds investigated. For these reasons, future investigations should be analyzed and tested in a wide population to introduce them in clinical practice.

## Data Availability

Data are available from corresponding author upon reasonable request.
